# RNA-Seq Analysis of Differentiated Keratinocytes Reveals a Massive Response to Late Events during Human Papillomavirus 16 Infection, Including Loss of Epithelial Barrier Function

**DOI:** 10.1128/JVI.01001-17

**Published:** 2017-11-30

**Authors:** T. Klymenko, Q. Gu, I. Herbert, A. Stevenson, V. Iliev, G. Watkins, C. Pollock, R. Bhatia, K. Cuschieri, P. Herzyk, D. Gatherer, S. V. Graham

**Affiliations:** a MRC—University of Glasgow Centre for Virus Research, Institute of Infection, Immunity and Inflammation, College of Medical, Veterinary and Life Sciences, University of Glasgow, Glasgow, United Kingdom; b HPV Research Group, University of Edinburgh, Edinburgh, United Kingdom; c Specialist Virology Centre, Royal Infirmary of Edinburgh, Edinburgh, United Kingdom; d Institute of Molecular Cell and Systems Biology, Glasgow Polyomics, University of Glasgow, Glasgow, United Kingdom; e Division of Biomedical & Life Sciences, Faculty of Health & Medicine, Lancaster University, Lancaster, United Kingdom; International Centre for Genetic Engineering and Biotechnology

**Keywords:** human papillomavirus 16, epithelial differentiation, keratinocyte transcriptome, cervical disease, epithelial cells

## Abstract

The human papillomavirus (HPV) replication cycle is tightly linked to epithelial cell differentiation. To examine HPV-associated changes in the keratinocyte transcriptome, RNAs isolated from undifferentiated and differentiated cell populations of normal, spontaneously immortalized keratinocytes (NIKS) and NIKS stably transfected with HPV16 episomal genomes (NIKS16) were compared using next-generation sequencing (RNA-Seq). HPV16 infection altered expression of 2,862 cellular genes. Next, to elucidate the role of keratinocyte gene expression in late events during the viral life cycle, RNA-Seq was carried out on triplicate differentiated populations of NIKS (uninfected) and NIKS16 (infected). Of the top 966 genes altered (>log_2_ = 1.8, 3.5-fold change), 670 genes were downregulated and 296 genes were upregulated. HPV downregulated many genes involved in epithelial barrier function, which involves structural resistance to the environment and immunity to infectious agents. For example, HPV infection repressed expression of the differentiated keratinocyte-specific pattern recognition receptor TLR7, the Langerhans cell chemoattractant CCL20, and proinflammatory cytokines interleukin 1α (IL-1α) and IL-1β. However, the type I interferon regulator IRF1, kappa interferon (IFN-κ), and viral restriction factors (IFIT1, -2, -3, and -5, OASL, CD74, and RTP4) were upregulated. HPV infection abrogated gene expression associated with the physical epithelial barrier, including keratinocyte cytoskeleton, intercellular junctions, and cell adhesion. Quantitative PCR (qRT-PCR) and Western blotting confirmed changes in expression of seven of the most significantly altered mRNAs. Expression of three genes showed statistically significant changes during cervical disease progression in clinical samples. Taken together, the data indicate that HPV infection manipulates the differentiating keratinocyte transcriptome to create an environment conducive to productive viral replication and egress.

**IMPORTANCE** HPV genome amplification and capsid formation take place in differentiated keratinocytes. The viral life cycle is intimately associated with host cell differentiation. Deep sequencing (RNA-Seq) of RNA from undifferentiated and differentiated uninfected and HPV16-positive keratinocytes showed that almost 3,000 genes were differentially expressed in keratinocytes due to HPV16 infection. Strikingly, the epithelial barrier function of differentiated keratinocytes, comprising keratinocyte immune function and cellular structure, was found to be disrupted. These data provide new insights into the virus-host interaction that is crucial for the production of infectious virus and reveal that HPV infection remodels keratinocytes for completion of the virus replication cycle.

## INTRODUCTION

Human papillomaviruses (HPVs) infect keratinocytes, causing mainly benign lesions or warts (1). Infection is usually transient and is cleared by the immune system (2). However, persistent infection with “high-risk” HPV genotypes (HR-HPV) can cause tumor progression to cervical (3), other anogenital (anal, penile, vulvar, and vaginal) (4), and oropharyngeal (5) cancers. In the case of the cervix, cervical intraepithelial neoplasia (CIN) generally precedes cervical cancer progression (6). CIN1 is thought to represent a transient HPV infection, while CIN3 represents clinically significant, persistent HPV infection that may, if left untreated, progress to cervical cancer (7).

The pathway of epithelial cell differentiation, from basal to granular layer, is tightly controlled by complex patterns of keratinocyte gene expression (8). The HPV infectious life cycle is tightly linked to epithelial differentiation. HPV infects basal epithelial cells, where it begins to express its genome. The viral replication factor E1 and its auxiliary protein, E2, which is also the viral transcription factor, together with the regulatory proteins E6 and E7 are expressed early in infection. E2, E6, and E7 have each been shown to control cellular gene expression (6). Viral gene expression required for vegetative viral genome amplification takes place in differentiating keratinocytes in the middle to upper epithelial layers (9). At this stage, other viral regulatory proteins, E4 and E5, that can regulate the host cell are expressed (6). Finally, L1 and L2 capsid protein synthesis and virion formation take place in granular layer keratinocytes, and virions are shed from the surface of the epithelium in dead squames (10). The epithelium presents a barrier to the environment and to infectious agents (11). Differentiated keratinocytes possess a dense filamentous network comprised of keratins and other molecules, such as filaggrin. Moreover, keratinocytes have an important role in innate and adaptive immunity, and cytokines, chemokines, and other immune signaling molecules released by these cells are essential for epithelial homeostasis (12). HPVs have evolved to modulate the epithelium to allow infection, virion formation, and egress (6), and many means by which HPV evades the immune response have been documented (13). Elucidating the interactions between HPV and the infected keratinocyte is key to understanding the HPV life cycle and how persistent infection may facilitate the development of cervical disease.

A number of previous studies have used a microarray approach to further our understanding of the HPV infectious life cycle and cancer progression. The first compared gene expression in normal keratinocytes with that in HPV31-infected keratinocytes (14). Two subsequent studies examined gene expression changes during tumor progression in HPV18-infected (15) or HPV33-infected (16) keratinocytes. A recent study investigated undifferentiated keratinocytes containing HPV16 or HPV18 episomal genomes. However, no studies have analyzed how cellular gene expression is altered in differentiating keratinocytes supporting the productive phase of the viral life cycle (17). Here we used next-generation sequencing (RNA-Seq) to examine global changes in the keratinocyte transcriptome due to epithelial differentiation and HPV infection. Our study reveals that HPV infection induces massive changes in the transcriptome during keratinocyte differentiation. In particular, changes led to the alteration of many genes encoding the keratinocyte structural barrier and immune function. Key statistically highly significant changes in gene expression were confirmed by reverse transcriptase quantitative PCR (qRT-PCR) and Western blotting and were investigated in clinical samples representing the cervical disease spectrum. These data can be used to understand late events in the viral life cycle and the mechanisms behind cervical disease progression.

## RESULTS

**FIG 1:**
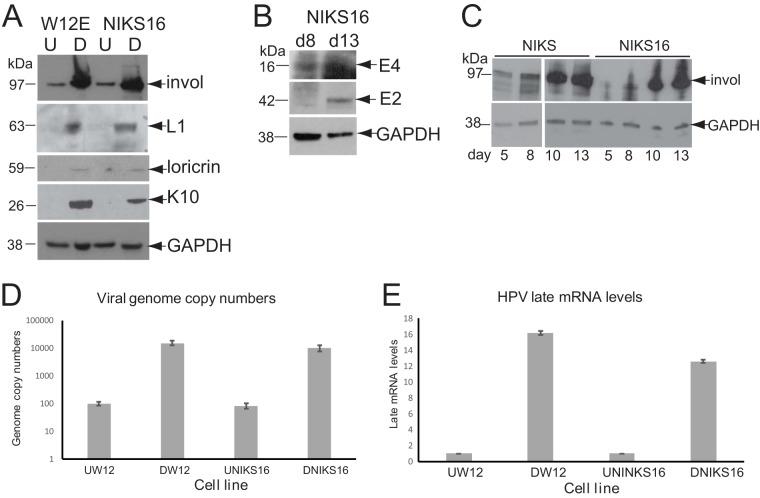
Characterization of the HPV16 life cycle in NIKS16 and W12 cells. (A) Expression levels of keratinocyte protein differentiation markers and viral L1 protein in undifferentiated (U = monolayer culture for 5 days) and differentiated (D = monolayer culture for 13 days) W12 and NIKS16 cells. GAPDH is shown as a loading control. (B) Expression levels of viral E2 and E4 proteins at 8 (mid-differentiation phase) and 13 (differentiated) days of a time course of NIKS16 differentiation in monolayer culture. (C) Time course of involucrin protein expression over a 13-day differentiation period (monolayer cells are mostly undifferentiated after 5 days of culture and fully differentiated after 13 days of culture) for NIKS and NIKS16 cells. invol, involucrin. (D) Absolute quantification by qPCR of L1 gene copies, as a measure of viral genomes, in differentiated W12 and NIKS16 cells. (E) Viral late mRNA levels quantified by detecting L1-containing mRNAs by qRT-PCR in undifferentiated and differentiated W12 and NIKS16 cells. Invol, involucrin; K10, keratin 10.

The HPV E2 transcription factor (18) and the viral oncoproteins E6 (19), E7 (20), and E5 (21) can all play a role in controlling cellular gene expression, and HPV infection is known to have a significant effect on keratinocyte growth and differentiation (6). In order to elucidate how cellular gene expression is altered during HPV infection, we examined changes in the keratinocyte transcriptome during differentiation and HPV16 infection using normal, spontaneously immortalized keratinocytes (NIKS) and the same cells stably transfected with HPV16 genomes (NIKS16). NIKS16 clone 2L maintains ∼100 episomal HPV16 genomes per cell (if cultured at low passage [<13]) and forms a CIN1-like (low-grade cervical disease) stratified epithelium upon raft culture, suggesting that these cells represent a transient HPV16 infection (22). We also examined a second HPV16 infection model, W12 cells, which are HPV16-infected basal cervical epithelial cells isolated from a patient with a low-grade cervical lesion (23). W12 clone 20863 (W12E) cells (if cultured at low passage [<17]) also maintain ∼100 episomal HPV16 genomes (24). Both cell lines are capable of differentiation. We used the differentiation protocol of Jeon et al. (24) in which cells are induced to differentiation by culturing to high density in 1.2 mM Ca^2+^. Differentiated NIKS16 and W12E cell populations expressed involucrin, loricrin, and keratin 10 proteins, key markers of keratinocyte differentiation (Fig. 1A). NIKS16 cells (and W12 cells [25]) expressed viral late proteins E2, E4, and L1 (Fig. 1A and B). A time course of NIKS and NIKS16 cell differentiation over a 13-day period is shown in Fig. 1C. As expected, NIKS cells (Fig. 1C, lanes 1 to 4) expressed more involucrin over the time course than NIKS16 cells (Fig. 1C, lanes 5 to 8) because HPV infection impairs epithelial differentiation (6). Absolute quantification of viral genome copies by PCR showed that differentiated W12 cells had an average of 15,250 genome copies, while there was an average of 9,937 copies of NIKS16 cells (Fig. 1D). Viral late mRNA levels, as measured by L1 open reading frame detection, the common reading frame in all late mRNAs, were increased 16.1-fold in W12 cells and 12.6-fold in NIKS16 cells upon differentiation (Fig. 1E). These data indicate that NIKS16 and W12 cells can be differentiated in monolayer cultures.

### Global changes in the transcriptome of HPV16-infected keratinocytes.

**FIG 2:**
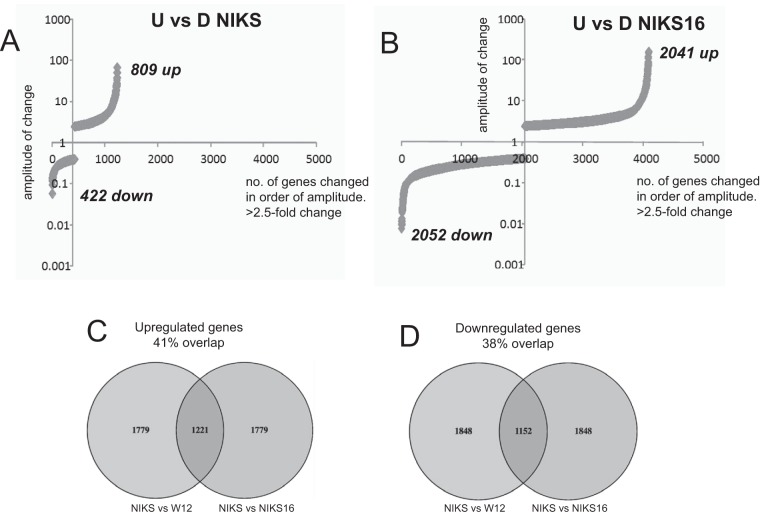
HPV16 infection induces massive changes in the keratinocyte transcriptome. (A) mRNA numbers expressed versus the level of expression of each individual mRNA in undifferentiated (U) versus differentiated (D) NIKS (HPV-negative) cells. (B) mRNA numbers expressed versus the level of expression of each individual mRNA in undifferentiated (U) versus differentiated (D) NIKS16 (HPV-positive) cells. (C) Venn diagram showing the percentage identity between upregulated genes of NIKS and NIKS16 cells and those of NIKS and W12 cells. (D) Venn diagram showing the percentage identity between downregulated genes of NIKS and NIKS16 cells and those of NIKS and W12 cells. Identity was determined using the GFOLD tool to calculate the differential fold changes of the two comparisons.

RNA-Seq was carried out using RNA prepared from undifferentiated and differentiated NIKS and NIKS16 populations. Comparing undifferentiated with differentiated uninfected NIKS, 809 mRNAs were upregulated while 422 mRNAs were downregulated (Fig. 2A). In contrast, in a comparison of undifferentiated to differentiated HPV16-infected NIKS16 cells, 2,041 genes were upregulated while 2,052 genes were downregulated (Fig. 2B). Because NIKS16 cells are derived directly from NIKS (22) and were differentiated using the same protocol, the 2,862 additional changes observed upon differentiation of HPV16-positive keratinocytes are likely attributable to HPV infection. A number of gene expression changes similar to that for NIKS16 cells was observed between undifferentiated and differentiated W12E cells (data not shown). There is no HPV-negative equivalent to W12 cells, but we examined the RNA-Seq changes in the transcriptome of differentiated W12 cells compared to NIKS cells and the RNA-Seq changes in NIKS16 cells compared to the parent NIKS cells. Despite the fact that these cells are of different origins, i.e., W12 is a female HPV-immortalized mucosal epithelial cell line and NIKS is a spontaneously male cutaneous epithelial cell line, there was a 41% overlap in upregulated genes (Fig. 2C) and a 38% overlap in downregulated genes (Fig. 2D). These data suggest that the effects of HPV infection and the differentiation process are somewhat similar for both cell types.

### HPV16 infection abrogates differentiation and epithelial barrier formation.

**FIG 3:**
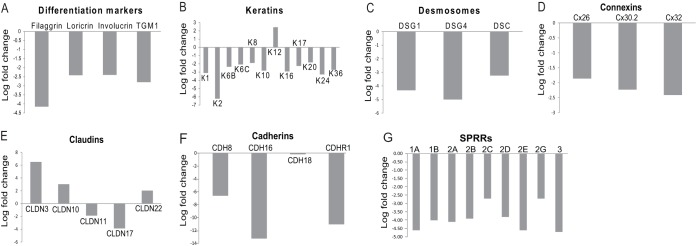
Keratinocyte differentiation and epithelial barrier function is altered by HPV infection. Significant changes in expression (>log_2_ = 1.8; 3.5-fold) of proteins involved in keratinocyte differentiation and epithelial barrier function comparing HPV16-infected, differentiated NIKS keratinocytes to uninfected, differentiated NIKS keratinocytes. These are mean values from three separate RNA-Seq experiments. (A) Markers of differentiation (filaggrin, loricrin, involucrin, and transglutaminase [TGM1]); (B) keratins (K); (C) desomosomal proteins, desmogleins (DSG) 1 and 4, and desmocoilin (DSC); (D) gap junction proteins, connexins (Cx) 26, 30.2, and 32; (E) claudins; (F) cadherins; (G) small proline-rich proteins (SPRRs).

We are interested in elucidating the link between keratinocyte differentiation and late events during HPV replication. Therefore, we compared the transcriptomes of differentiated NIKS and NIKS16 cells. Three replicate, single-end sequencing experiments were carried out, and changes that gave a *P* value of >0.05 across three replicates were discarded to achieve significance. Table S1 in the supplemental material lists the top 966 changes in gene expression (*P* < 0.05, log_2_ > 1.8, 3.5-fold change). There were 670 downregulated genes, while 296 were upregulated, with a range of 184-fold downregulated to 87-fold upregulated. The data in Fig. 3 show the mean of the results of three separate RNA-Seq experiments. As expected, key epithelial differentiation markers were downregulated in NIKS16 cells (Fig. 3A). Suprabasal layer keratins were also downregulated. Keratin 12, which is usually expressed only in the corneal epithelium (26), was the only keratin whose levels were increased in NIKS16 cells (Fig. 3B). Expression of cell junction proteins that are key to epithelial barrier function was significantly altered. Desmosome cell-cell junction proteins required for cell adhesion (Fig. 3C) (27), and gap junction connexin (Cx) proteins 26, 30, and 32, which allow transfer of small molecules between differentiating epithelial cells (28), were downregulated (Fig. 3D). Claudin proteins control tight junctions, and CLDN3, -10, and -22 were upregulated while CLDN11 and -17 were downregulated (Fig. 3E). Claudin upregulation can still have a negative impact on the function of tight junctions in a phenomenon referred to as “leaky claudins” (29). Several adherens junction-associated cadherins (27) were also downregulated (Fig. 3F). Small proline-rich repeat protein (SPRR) family members that contribute to barrier formation by forming the cornified layer in differentiated epithelial cells (30) were downregulated (Fig. 3G). The calcium gradient in the epithelium is altered upon loss of barrier formation (31), and levels of RNAs encoding a range of calcium ion-binding proteins (e.g., S100A8/A9 calgranulin complex, DSG1, matrix Gla protein [MGP], and calcium/calmodulin kinase 2B [CAMK2B]) were reduced (data not shown). Taken together, the data suggest that HPV infection inhibits epithelial barrier formation and epithelial integrity.

The epithelial barrier also involves immune signaling, and significant changes in expression of many genes whose products are involved in intrinsic and innate immunity were also observed (Table 1). Previously, a microarray study revealed that HR-HPV repressed activation of the immune response in undifferentiated epithelial cells through interleukin 1β (IL-1β). Similarly, in HPV-infected differentiated cells, we found that *IL1B* gene expression was downregulated. *IL1A* was also downregulated, as were *IL32G* and *IL36B*, which activate keratinocyte immune functions. The Langerhans cell chemoattractant CCL20 was downregulated in the presence of HPV16. However, CCL28 that controls T-cell homing in mucosal epithelia, E6/E7-regulated CXCL12, and CX3CL1 were all upregulated. The type I interferon (IFN) regulator, IRF1, and the epithelial IFN-κ were upregulated, an unexpected finding since HPV E6 and E7 have been shown to inhibit their expression (32–34). We found a 6-fold downregulation of the viral DNA pattern recognition receptor TLR7, which is expressed specifically in differentiated keratinocytes (35), together with upregulation of viral restriction factors APOBEC3B, IFIT1, -2, -3, and -5, CD74, OASL, and RTP4 (Table 1). These data indicate that the keratinocyte-mediated immune response is under the control of HPV16 in the upper epithelial layers and that there are significant differences to HPV regulation of immune signaling between differentiated and basal epithelial cells (17).

**TABLE 1:** Changes in expression of immune regulatory molecules and viral restriction factors

Gene	Category^*a*^	Negative fold change	Positive fold change
*TLR7*	PRR	6-fold	
*NLRP3*	Inflammasome component	7-fold	
*IL1A*	Cytokine	7-fold	
*IL1B*	Cytokine	4-fold	
*IL32G*	Cytokine	17-fold	
*IL36B*	Cytokine	6-fold	
*CCL20*	Chemokine	7-fold	
*CCL28*	Chemokine		5-fold
*CXCL12*	Chemokine		4-fold
*CX3CL1*	Chemokine		32-fold
*APOBEC3C*	Restriction factor		4-fold
*IFIT1*	Restriction factor		6-fold
*IFIT2*	Restriction factor		7-fold
*IFIT3*	Restriction factor		7-fold
*IFIT5*	Restriction factor		4-fold
*CD74*	Restriction factor		4-fold
*OASL*	Restriction factor		4-fold
*RTP4*	Restriction factor		13-fold
*IRF1*	IFN regulatory transcription factor		4-fold
*IFN*κ	Interferon kappa		8-fold

a PRR, pattern recognition receptor; IFN, interferon.

### Cellular networks involved in the immune response and keratinocyte structure and metabolism are altered by HPV16 infection.

**FIG 4:**
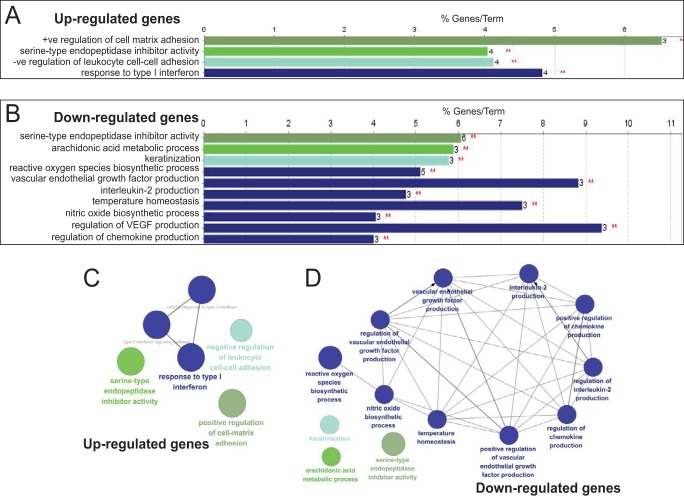
ClueGO analysis of significantly up- and downregulated genes in HPV16-infected, differentiated NIKS keratinocytes compared to uninfected, differentiated NIKS keratinocytes. We used CluePedia, which extends ClueGO (71) functionality down to genes and visualizes the statistical dependencies (correlation) for markers of interest from the experimental data. (A) Gene ontology (GO) pathway terms specific for upregulated genes. (B) GO pathway terms specific for downregulated genes. The bars represent the numbers of genes associated with the terms on the left. The percentage of altered genes is shown above each bar. Red asterisks refer to significance. (C) Functionally grouped network for upregulated genes. (D) Functionally grouped networks for downregulated genes. Only the label of the most significant term per group is shown. The size of the nodes reflects the degree of enrichment of the terms. The network was automatically laid out using the organic layout algorithm in Cytoscape. Only functional groups represented by their most significant term were visualized in the network. *P*_adj_ changes of <0.05 were analyzed.

**FIG 5:**
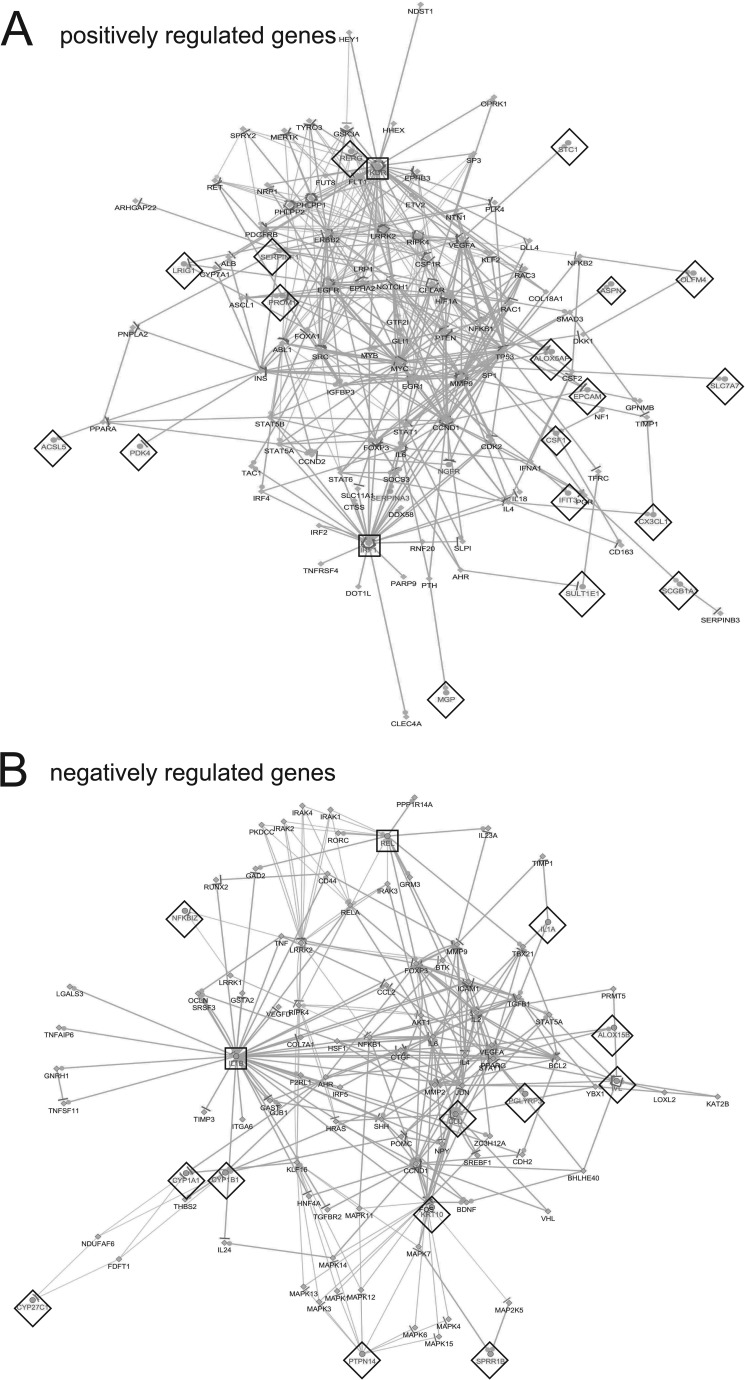
Interactome of negatively and positively changed genes comparing differentiated NIKS with differentiated NIKS16 cells. Interactome of genes linked through statistical correlation of upregulated (A) and downregulated (B) genes from the experimental data (*P* < 0.05). Gray lettering and diamonds indicate genes identified in the RNA-Seq data set. Black lettering indicates linked genes. Nodes for genes identified in the data set are indicated by black box outlines. Dots/lines surrounding nodes indicate the numbers of linked pathways. The pathway analysis was produced using CluePedia (http://apps.cytoscape.org/apps/cluepedia).

Following adjustment of the data set to exclude any changes where the triplicate values gave a *P* value of >0.05, gene ontology network pathway analysis of the top 1,000 up- or downregulated genes was carried out. Analysis revealed distinct gene classes whose expression was altered by HPV16 infection (Fig. 4). Response to type I interferon was upregulated, but cytokine and chemokine expression was repressed. Cell matrix adhesion was upregulated, while cell-cell adhesion was downregulated (reported by the Cytoscape program as negative regulation of upregulated leukocyte genes) (Fig. 4A). Other significantly downregulated pathways included keratinization, arachidonic acid metabolism, reactive oxygen and nitric oxide biosynthesis, vascular endothelial growth factor (VEGF), and temperature homeostasis (Fig. 4B). Network analysis indicated that pathways related to the type I interferon response were strongly connected (Fig. 4C), while downregulated genes were associated through cytokine/chemokine/VEGF pathways (Fig. 4D). A log_2_ change value of >2.5 was chosen to construct a wider pathway linkage diagram. IRF1 and KDR genes were major HPV-upregulated genes encoding hub proteins that connected a number of cell growth and apoptosis signaling pathways. IL-1β and REL, an NF-κB family transcriptional coactivator, linked HPV-downregulated cytokine and VEGF (Fig. 5).

### Verification of gene expression changes due to HPV16 infection.

Six genes from among those with the statistically most highly significant changes in expression (Table 2) (adjusted *P* [*P*_adj_] values are shown where a *P* of 0.05 in the triplicate data set is given the value of 1) were selected for further study (negative, *DSG1*, *SERPINB3*, and *KRT10*; positive, *VTCN1*, *KDR*, and *AZGP1*). Although *IL1B* had a *P*_adj_ of 1 (actual *P* value = 0.05), it was also included because expression of this important gene was found to be a key gene network hub in both undifferentiated (17) and differentiated HPV-infected cells (Fig. 5). These genes all encode proteins with known metabolic or immune/inflammatory roles in the normal epithelium. KRT10 is a differentiation-specific keratinocyte filament protein. DSG1 is a calcium-binding desmosome regulator. KDR (vascular endothelial growth factor receptor 2 [VEGFR-2]) has an autocrine function in cell proliferation, adhesion, and migration (36). IL-1β “node” cytokine activates adaptive immunity. VTCN1 is a T-cell activation inhibitor. SERPINB3 controls epithelial inflammatory responses, and AZGP1 is induced by IFN-γ in keratinocytes (37). mRNA expression in NIKS versus NIK16 cells and W12 cells was validated by qRT-PCR (Table 2).

**TABLE 2:** RNA-Seq expression changes in mRNAs of statistical significance (*P* <0.025) verified by qRT-PCR

Gene	*P*_adj_	NIKS16/NIKS fold change by RNA-Seq	NIKS16/NIKS fold change by qPCR	W12/NIKS fold change qPCR	Gene product/function
*DSG1*	2.05 × 10^−5^	−19.95	−4.20	−3.52	Desmoglein 1: calcium-binding desmosome regulator
*IL1B*	1	−8.68	−5.65	−7.73	Interleukin 1β: inflammatory response regulator
*SERPINB3*	0.008	−8.40	−4.28	−4.410	Intracellular protease inhibitor, inhibits active inflammatory response
*KRT10*	0.021	−7.07	−10.26	−3.70	Keratin 10: epithelial cytofilament
*KDR*	0.025	10.21	10.10	4.30	VEGFR-2, tyrosine kinase receptor
*VTCN1*	1.4 × 10^−9^	46.12	8.94	10.56	V-set domain-containing T-cell activation inhibitor-1
*AZGP1*	2.05 × 10^−5^	12.64	7.49	8.31	Zinc alpha-2 glycoprotein: lipid metabolism
*GAPDH*		1	1	1	Glyceraldehyde-3-phosphate dehydrogenase (control)
*Beta-actin*		1	1	1	Actin (control)

**FIG 6:**
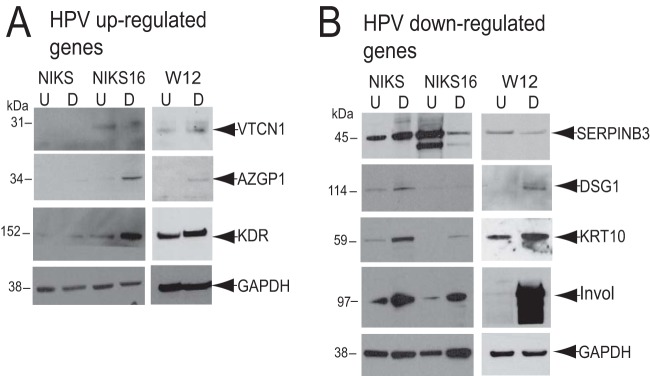
Western blot analysis of protein levels encoded by selected, significantly altered mRNAs (Table 2). Protein extracts were prepared from undifferentiated (U) and differentiated (D) HPV-negative NIKS and HPV16-positive NIKS16 and W12 cell populations. Much greater levels of involucrin (invol) were detected in the differentiated than in the undifferentiated cell populations, indicating that differentiation was achieved. GAPDH was used as a protein loading control. (A) Protein levels corresponding to significantly upregulated mRNAs; (B) protein levels corresponding to significantly downregulated mRNAs.

Protein levels encoded by these mRNAs were examined in undifferentiated and differentiated NIKS, NIKS16, and W12 cells (Fig. 6). Levels of AZGP1, KDR, DSG1, KRT10, and involucrin increased upon NIKS16 and W12 cell differentiation, while SERPINB3 levels decreased and VTCN1 levels did not change. There were higher levels of VTCN1, AZGP1, and KDR in NIKS16 cells than in differentiated NIKS cells, but KRT10 and DSG1 levels were much lower in differentiated NIKS16 cells than in NIKS cells, as expected. SERPINB3 levels were greatly reduced following differentiation of NIKS16, but not NIKS, cells. There is no HPV-negative W12 cell equivalent to NIKS cells, so it is not possible to be sure if the changes in protein expression in W12 cells upon differentiation are due to HPV infection. These data confirm that selected keratinocyte transcriptomic changes due to HPV16 infection are reflected in protein levels.

### HPV16 infection-regulated mRNAs as biomarkers of cervical disease.

**FIG 7:**
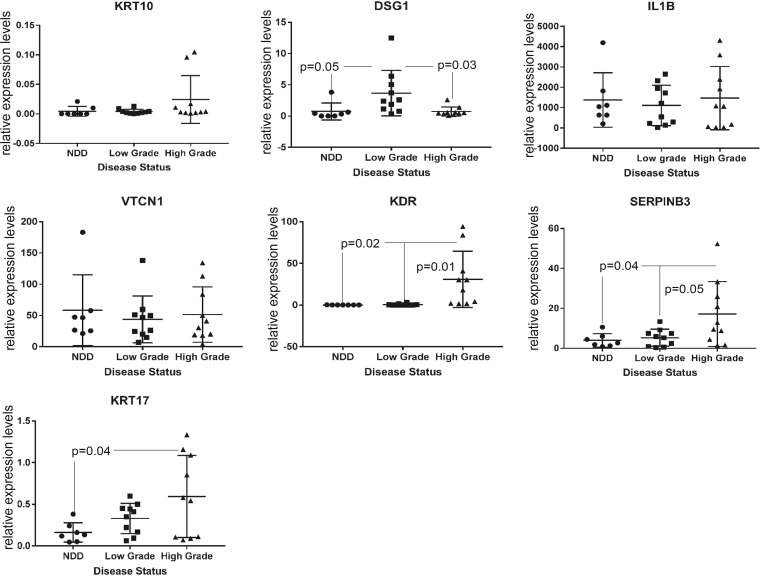
Expression levels of selected, significantly altered mRNAs in different grades of HPV-associated preneoplastic cervical disease. mRNA expression levels were calculated from qRT-PCR data using GAPDH and beta-actin as the internal controls and expressed relative to levels in a single sample of differentiated, HPV16-positive W12 cell RNA that was included in every PCR run. NDD, no detectable disease/borderline, all HPV negative; low-grade disease, cervical intraepithelial neoplasia 1 (CIN1), all HPV positive; high-grade disease, cervical intraepithelial neoplasia 1 (CIN3), all HPV positive.

It could be argued that the NIKS16 model of the HPV16 life cycle may not directly relate to cervical HPV infection because NIKS16 cells are foreskin, not cervical, keratinocytes, However, NIKS16 cells appeared to represent a low-grade cervical lesion when grown in raft culture (22), and there is a similar organization of the HPV life cycle at different anatomical sites (38). HPV16-associated gene expression changes in keratinocytes could be related to the productive life cycle but could equally be associated with cervical disease progression. Therefore, to test whether any of the HPV-related changes in keratinocyte gene expression that we detected could have potential as HPV-associated cervical disease biomarkers, we quantified levels of expression of three upregulated and three downregulated genes (encoding two regulators of the inflammatory response [IL-1β, SERPINB3], two proteins involved in cell signaling [KDR, VTCN1], and two involved in barrier function [KRT10, DSG]) by qRT-PCR in liquid-based cytology (LBC; Pap smear) samples. Apart from choice due to gene function, IL-1β RNA was chosen for analysis because it encoded a hub in the interactome (Fig. 5), VTCN1 and DSG1 were chosen as representatives of very highly significantly altered RNAs, KRT10 was chosen as a differentiation marker, KDR was chosen as an RNA potentially involved in cancer formation, and SERPINB3 was chosen because it was an early identified cervical cancer marker (39). Due to lack of mRNA, we were unable to test AZGP1. A control cDNA from differentiated W12E cervical keratinocytes was included in each qRT-PCR plate as a standard, and absolute levels of RNA in the LBC samples (normalized against GAPDH [glyceraldehyde-3-phosphate dehydrogenase]) were calculated using the Pfaffl standard curve method (40, 41). KRT17 was analyzed as a known biomarker of cervical disease progression (42). Figure 7 shows the mean and range of values for each mRNA in 7 samples of no detectable disease (NDD), 10 samples of low-grade cervical lesions (CIN1), and 10 samples of high-grade cervical lesions (CIN3). Although we analyzed 10 samples graded as NDD, once HPV typing status was revealed, 3 of these 10 were HPV positive. We decided to exclude these from the analysis in order to compare HPV-negative and HPV-positive clinical samples. KRT10 mRNA levels were very low, making analysis of significance difficult, and there was high variability in levels of IL-1β and VTCN1. However, very high levels of IL-1β mRNA were detected in all patient samples, regardless of disease stage. DSG1 was significantly increased between no detectable disease (NDD) samples and low-grade disease samples but significantly decreased between low-grade and high-grade disease samples. KDR and SERPINB3 levels were significantly upregulated between low-grade and high-grade disease samples, similar to the positive control, KRT17. These data suggest that RNA-Seq analysis has the potential to uncover novel biomarkers of cervical disease.

## DISCUSSION

The aim of our work was to examine how human papillomavirus replication is linked to keratinocyte differentiation. In particular, we were interested in how differentiating keratinocytes respond to HPV infection during the late, productive phase of the viral life cycle. As a model to compare HPV-negative to HPV-positive keratinocytes, we used NIKS and NIKS16 cells. NIKS are spontaneously immortalized neonatal foreskin keratinocytes that have no alterations in differentiation or apoptosis (43). NIKS16 cells were derived directly from NIKS cells by stable transfection of the HPV16 genome isolated from W12 cells (22). We have shown that the NIKS16 cells adequately support the infectious viral life cycle (as previously reported [22]) because several key markers of keratinocyte differentiation and viral life cycle completion, i.e., viral genome amplification, viral late mRNA induction, and capsid protein production, were detected. Moreover, because there was repression of VEGF pathways, reduced expression of HOX and MMP proteins, and no general upregulation of EMT markers, these cells are likely not undergoing tumor progression. Because NIKS cells are foreskin keratinocytes, they will likely have a number of differences in their gene expression profile compared to that of cervical keratinocytes. We did not have access to spontaneously immortalized HPV-negative cervical keratinocytes, but we compared changes in W12 gene expression with NIKS cells. There was around 40% identity in the up- and downregulated genes between NIKS16 and W12 cells. W12 cells are naturally infected, female, mucosal epithelial cells, while NIKS16 cells are male cutaneous epithelial cells and spontaneously immortalized, and these significant differences likely account for the remaining 60% of nonoverlapping genes. Therefore, NIKS16 is potentially a more robust model for HPV16-associated penile lesions than cervical lesions, and it will be interesting in the future to compare these data sets with similar sets from differentiated uninfected and infected cervical keratinocytes. Three-dimensional raft culture would undoubtedly provide a superior approach for examining keratinocyte differentiation and HPV infection. However, for analysis of late events in the viral life cycle in differentiated keratinocytes, this is technically challenging and difficult to reproduce, because RNA isolation from multiple, microdissected, upper epithelial layer sections would be required for triplicate RNA-Seq experiments. Our current data set should provide an important basis for subsequent analysis of raft culture models.

Many transcriptomic studies have analyzed cellular changes during HPV-associated tumor progression or due to overexpression of viral proteins (14, 15, 18, 21, 44–51). Of the microarray studies investigating changes due to HPV infection, as opposed to tumorigenesis, one compared expression of HPV31-positive and -negative cervical keratinocytes (14), a second examined HPV33-negative and -positive vaginal keratinocytes (16), while another compared undifferentiated anogenital keratinocytes with or without episomal HPV16 and HPV18 genomes (17). All of these studies focused on the effect of HPV on basal keratinocytes, the site of viral entry, and initial replication. No studies to date have examined keratinocyte responses to late events in the viral replication cycle. Moreover, the previous studies used microarray analysis, which does not provide the unparalleled depth of information available from RNA-Seq. To our knowledge, this is the first report comparing the transcriptomes of uninfected and HPV-infected differentiated keratinocytes using RNA-Seq. HPV infection induced massive changes (2,862 additional expression changes compared to the transcriptome of HPV-negative NIKS cells) in the keratinocyte transcriptome. Desmosomes, adherens, and tight and gap junction classes were all downregulated in the presence of HPV16, likely due to HPV16 E6/E7 reactivation of the cell cycle and decreased keratinocyte differentiation (52), as has been reported previously (17). Together with high-level downregulation of SPRRs, altered arachidonic acid metabolism, and changes in mucins, one can conclude that HR-HPV infection results in a broad abrogation of epithelial barrier function and epithelial integrity. Reduced barrier function could result in increased fragility of cells in the upper epithelial layers to facilitate viral egress.

Keratinocytes are key players in the immune response, and they produce a panoply of molecules involved in host defense against pathogens. In differentiated NIKS16 keratinocytes, HPV infection altered gene expression related to innate immunity, including reduced expression of the *TLR7*, *IL1A*, *IL1B*, *NLRP3*, *IL36B*, and *IL32G* genes. TLR7, a pattern recognition receptor for viral nucleic acids, is upregulated upon keratinocyte differentiation (35) and activates proinflammatory cytokines and other molecules involved in the adaptive immune response. There was a 6-fold downregulation of TLR7 in the presence of HPV16, suggesting that the virus represses pattern recognition during vegetative viral genome amplification, but by a different mechanism than that used in undifferentiated keratinocytes where infection suppresses TLR9 (17). There was a corresponding reduction in NF-κB-regulated CCL20, known to be regulated by HPV E7 (53) and required to recruit Langerhans cells. Indeed, NF-κB signaling was affected, and the NF-κB family member REL was a major HPV-regulated control node in the pathway analysis of negatively regulated genes (Fig. 5). Surprisingly, we discovered that the epithelium-specific IFN-κ, and IRF1 that controls type I IFNs, was upregulated by HPV16 in differentiated keratinocytes. Previously, HPV16 E7 or HPV38 E6/E7 were shown to inhibit IRF1 expression (32, 33), while HPV16 E6 was shown to repress IFN-κ transcription through promoter methylation (54). However, these studies used overexpression of the viral oncoproteins. The levels of E6 or E7 proteins may be much lower in differentiated keratinocytes than in the undifferentiated epithelial cells or cervical cancer cells used in these studies. In contrast to E6 and E7, E5 can stimulate IRF1 expression in HaCaT cells (55). Changes due to expression of the entire virus genome may be more complex and quite different from that seen with the expression of individual viral proteins. Upregulation of interferon-induced protein with tertatricopeptide repeats (IFITs) corresponded with the observed activation of the type I interferon response. Only IFIT1 has been shown to inhibit HPV replication (56, 57); therefore, the roles of other IFITs in inhibiting HPV infection remain to be determined. APOBEC3B was upregulated; however, we found no changes in expression of APOBEC3A, a known HPV restriction factor, but its expression may be differentially regulated only in less differentiated keratinocytes (58). The observed upregulation of CXCR6 and CXCL12 is in agreement with CXCL12 detection in HPV-induced lesions and its role in the productive HPV life cycle (59). We also detected changes in some SERPINs (e.g., SERPINB3) that are involved in the inflammatory/immune response (60). We did not detect changes in STAT1, which has been shown to be controlled by E6 and E7 (61). It is possible that it undergoes changes of less than the cutoff of >3.5-fold considered here. However, STAT1 controls IRF1 expression, which was upregulated 4-fold, and STAT1 was a central node connecting gene pathways regulated by HPV16 (Fig. S1). Of course, because we used an immortal cell line, immortalization could account for some of the changes we observed. It will be important to analyze innate immune regulators in differentiated primary cervical keratinocytes in future studies. Our data reveal that HPV suppression of intrinsic and innate immunity takes place not only in infected basal epithelial cells (17) but also in keratinocytes harboring late events in the HPV life cycle and that a differentiation stage-specific set of events may be relevant to this life cycle stage. The stimulation of the IFN response and viral restriction factors in differentiated HPV-infected cells requires further study. Production of progeny viral genomes and virions may stimulate the IFN response and lead to apoptosis, and this could aid release and dissemination of virus particles.

The E5, E6, E7, and E2 proteins of HPV16 are known to control cellular gene expression. E6 and E7 control keratinocyte cell cycle and apoptosis and abrogate differentiation. Many of the changes in gene expression that we have observed can be attributed to these functions of the viral oncoproteins. These changes are clearly important for the replicative life cycle of HPV16 but could also contribute to HPV persistence and development of neoplasia (6). Similar to data from one overexpression study of HPV16 E6 in human foreskin keratinocytes (50), the differentiation marker involucrin, vimentin, which is expressed upon epithelial stress, and signal transduction proteins MEST and H19 were upregulated in our analysis. However, we detected none of the other changes affecting cell cycle, proliferation, DNA damage, metabolism, or signaling that have previously been reported (50). We discovered only seven genes (those encoding semaphorin 5A [SEMA5A], CXCL1, ENTPOT, follistatir [FST], cytochrome P450 [CYP] 24A1, pleckstrin homology-like domain A1 [PHLDA1], and ribosomal protein S27-like [RPS27L]) out of a total of 99 altered in another study using small interfering RNA (siRNA) depletion of E6 in HPV-positive tumor cells (47). Compared to a study of W12 cells with integrated HPV16 genomes expressing different levels of E6 and E7, we detected genes encoding E6-regulated loricrin (LOR) and cytochrome P450 (CYP) 1B1 and E7-regulated FABP4, SERPINA3, and SLURP1 out of the top 20 genes upregulated by each protein (62). Only 1 out of 12 master regulators of E6 or E7 function defined by Smith et al. (62) was in common with our study. This was downregulation of PRDM1 (BLIMP-1), which acts as a repressor of IFN-β gene expression. E5 overexpression in HaCaT keratinocytes yielded 61 mRNAs with significant changes (21), but only two of these (keratin 8 and MMP16 mRNAs) were in common with our RNA-Seq data. In a microarray study of E2 overexpression in U2OS cancer cells where 74 genes were found to be regulated, only 3 of these (those encoding heterotrimeric G-complex protein 11 [GNG11] involved in cell signaling, histamine *N*-methyltransferase [HNMT] involved in methylation of histamine, and SERPINA3, which is upregulated in response to decreased transglutaminase activity) were altered in our study. The increased viral oncoprotein expression levels in HPV-positive cancer cells, or in cells overexpressing viral proteins, compared to the model we have used, i.e., keratinocytes supporting expression of all viral proteins from the intact HPV16 genome where expression levels are much lower (3), could explain the fact that we did not detect many of these changes. Moreover, we have only considered expression changes of >3.5-fold, while these other studies considered 2-fold changes. RNA-Seq analysis of the W12 tumor progression series (63) would help to delineate infection- versus cancer-related changes.

Liquid-based cytology samples (LBCs; Pap smear samples) contain cells scraped from the top of the cervical epithelium and thus contain HPV-infected differentiated keratinocytes. Therefore, some of the mRNA changes we have detailed could be biomarkers of cervical disease. Very high levels of IL-1β mRNA were detected in all patient samples, regardless of disease stage, likely due to inflammation commonly observed in the diseased cervix. Statistically significant changes in KDR and SERPINB3 expression, like the known biomarker KRT17, indicate their potential in identifying high-grade cervical disease. DSG1 was significantly increased between no-detectable-disease (NDD) samples and low-grade disease samples but significantly decreased between low-grade and high-grade disease samples. This is in contrast to the clear downregulation of DSG1 expression due to HPV16 infection of NIKS and suggests either that NIKS16 cells may not represent a low-grade HPV16-positive lesion or that the levels of DSG1 in cervical keratinocytes are very different from those in foreskin keratinocytes.

In conclusion, we report for the first time RNA-Seq analysis of changes in the keratinocyte transcriptome caused by HR-HPV infection. Infection caused massive changes in epithelial gene expression. These changes showed mainly a profile expected of viral infection rather than tumor progression. The large data set we have developed opens up the possibility of a deeper understanding of late events in the HPV replication cycle in response to keratinocyte differentiation. As well as shedding light on late events during the HPV16 life cycle, the RNA-Seq data might uncover potential biomarkers of HPV-associated anogenital disease progression. From our analysis, DSG1, KDR, and SERPINB3 expression may have potential as robust markers that can risk-stratify cervical disease, i.e., identify cervical disease cases that have a high probability of regression, and this would be of significant clinical value. However, further longitudinal studies in which biomarker status is linked to clinical outcomes are required to validate any biomarkers for such an application.

## MATERIALS AND METHODS

### Clinical sample panel underlying pathology and HPV status.

Anonymized, cervical liquid-based cytology samples were obtained from the Scottish National HPV Archive, which holds Generic Scotland A Research Ethics Committee approval for Research Tissue Banks (REC Ref 11/AL/0174) for provision of samples for HPV-related research after approval from an independent steering committee. The Scottish HPV Archive also comes under the auspices of the NHS Lothian Bioresource. The panel comprised HPV-negative/cytology-negative (no disease, *n* = 7) samples with low-grade cytological abnormalities with histological confirmation of cervical intraepithelial neoplasia 1 (CIN1) (low-grade disease, *n* = 10) and samples with high-grade cytological abnormalities with histological confirmation of CIN2 or worse, including cancer (high-grade disease, *n* = 10). Cytology grades were reported according to the British Society for Clinical Cytopathology (BSCC) classification (64–66). HPV testing was performed by the Optiplex HPV genotyping assay (Diamex, Heidelberg, Germany) according to the manufacturer's instructions. The Optiplex test is a PCR-based assay which uses a Luminex platform for the detection of 24 individual HPV types, including all types established as high risk according to the International Agency on Research on Cancer. For the purposes of this panel, the main function of the genotyping was for the annotation of no-disease “controls.” Women with negative cytology and HPV-negative status are at a very low risk of underlying disease (negative predictive value for a high-grade lesion of >95% [67]) All experiments were performed in compliance with relevant laws and institutional guidelines and in accordance with the ethical standards of the Declaration of Helsinki.

### Cell lines.

W12E (24), NIKS (43), and NIKS16 (22) cells were cocultured in E-medium with mitomycin C-treated J2 3T3 fibroblast feeder cells as previously described (24). Differentiation was induced by growth to high density in 1.2 mM Ca^2+^ (24). 3T3 cells were grown in Dulbecco's modified Eagle medium (DMEM) with 10% donor calf serum. Prior to harvesting, 3T3 cells were removed by trypsinization, and cell layers were washed twice with phosphate-buffered saline (PBS). All cells were maintained under humidified 5% CO_2_–95% air at 37°C.

### RNA isolation. (i) Cell lines.

Protocols followed the manufacturer's instructions. Total RNA was prepared using a Qiagen RNeasy kit. RNA was quantified and purity assessed by measuring the ratio of the absorbance at 260 to that at 280 nm using a Nanodrop ND-1000 spectrophotometer (ThermoScientific). Polyadenylated RNA was prepared using an oligo(dT)-based mRNA extraction kit (Oligotex, Qiagen).

### (ii) Clinical samples.

LBC cells in 4 ml of PreservCyt collection medium (Cytyc Corporation) were pelleted by centrifugation in a Beckman GPR benchtop centrifuge at 1,500 × *g* for 10 min. The cell pellet was washed with sterile PBS. RNA extraction was carried out using an RNeasy miRNA preparation kit (Qiagen). RNA was quantified and purity was assessed by measuring the ratio of the absorbance at 260 to that at 280 nm using a Nanodrop ND-1000 spectrophotometer.

### qRT-PCR.

For cell line and clinical samples, DNA was removed using Maxima DNase, and treated RNA was reverse transcribed using a Maxima first-strand cDNA synthesis kit according to the manufacturer's instructions (ThermoScientific). Standard curves were generated as recommended (Applied Biosystems instruction manual). Triplicate amplification reactions containing 100 ng cDNA each were carried out. GAPDH and β-actin were used as the internal standard controls. Probes and primers were as follows: GAPDH F, 5′-GAAGGTGAAGGTCGGAGT-3′; GAPDH R, 5′-GAAGATGGTGATGGGATTTC-3′; GAPDH probe, 5′-CAAGCTTCGTTCTCAGCC; KRT10F, 5′-TGGTTCTTGCCTCAGAAGAGCTGA-3′; KRT10 R, 5′-AGTACACGGTGGTGTCTGTGTCAT-3′; KRT10 probe, TGTGTCCACTGGTGATGGGAATGTGG-3′; DSG1 F, 5′-ACGTTCACGATAACCGACCAGCAT-3′; DSG1 R, 5′-ATTCCATGCAAATCACGGCCAGAG-3′; DSG1 probe, 5′-AACGTGGTAGTGACAGAGAGAGTGGT-3′; KDR F, 5′-TGGTTCTTGCCTCAGAAGAGCTGA-3′; KDR R, 5′-AGTACACGGTGGTGTCTGTGTCAT-3′; KDR probe, 5′-TGGCATCTGAAAGCTCAAACCAGACA-3′; IL1B F, 5′-TCTGTACCTGTCCTGCGTGTTGAA-3′; IL1B R, 5′-TGCTTGAGAGGTGCTGATGTACCA-3′; IL1B probe, 5′-CAAGCTGGAATTTGAGTCTGCCCAGT-3′; VTCN1 F, 5′-CACCAGGATAACATCTCTCAGTGAA-3′; VTCN1 R, 5′-TGGCTTGCAGGGTAGAATGA-3′; VTCN1 probe, 5′-AAGCTGAAGATAATCCCATCAGGCAT-3′; SERPINB3 F, 5′-GCTGCCAAATGAAATCGATGGTCTCC-3′; SERPINB3 R, 5′-TTCCCATGGTTCTCAACGTGTCCT-3′; SERPINB3 probe, 5′-AACTCGGTTCAAAGTGGAAGAGAGCT-3′; KRT17 F, 5′-GATGCGTGACCAGTATGAGAAG-3′; KRT17 R, 5′-CGGTTCAGTTCCTCTGTCTTG-3′; KRT17 probe, 5′-ATGGCAGAGAAGAACCGCAAGGAT-3′. Reaction mixtures (25 μl) contained 1× Mastermix (Stratagene), 900 nM primers, 100 nM probe, and 300 nM reference dye (Stratagene). qRT-PCRs were performed and analyzed on an Applied Biosystems 7500 Fast System. Graphing and statistical analyses were performed using GraphPad Prism 7. Statistical analysis (all three groups were compared to each other) was performed by the Kruskal-Wallis test, and data were analyzed by one-way analysis of variance (ANOVA) with Tukey's posttest. A significance level at a *P* of <0.05 was used.

### Western blot analysis.

Cells were lysed in 2× protein loading buffer (125 mM Tris [pH 6.8], 4% SDS, 20% glycerol, 10% mercaptoethanol, and 0.006% bromophenol blue with fresh protein inhibitor cocktail [Roche, United Kingdom]). Protein extracts were passaged with a syringe through a 22-gauge needle 15 times and then sonicated in a Sonibath for three 30-s pulses to break up the DNA strands. The samples were boiled at 100°C for 5 min before being loaded on a 12% Novex gel (Invitrogen) and electrophoresed at 150 V for 1 h in 1× MES (morpholineethanesulfonic acid) buffer. Gels were transferred to a nitrocellulose membrane using an iBlot transfer kit and iBlot gel transfer stacks (Invitrogen) per the manufacturer's instructions. Membranes were blocked in 5% milk powder in PBST (PBS-Tween 20) at room temperature for at least 1 h. Membranes were washed three times in PBST for 5 min each and then incubated with primary antibody. Mouse GAPDH (Meridian; 6C5), involucrin (Sigma; I9018), loricirn (Abcam; ab85679), serpinB3 (Sigma; 2F5), and keratin 10 (Abcam; ab9026) monoclonal antibodies were used at a dilution of 1:1,000. HPV16 E2 antibody (Santa Cruz; TVG261) was used at 1:500 dilution. HPV16 L1 antibody (Dako; K1H8) was used at a 1:400 dilution. HPV16 E4 antibody (gift of J. Doorbar, Cambridge, United Kingdom; clone B11) was used at a dilution of 1:50. Rabbit DSG1 (Abcam; ab133662), VEGFR2 (KDR) (Abcam; ab39256), and AZGP1 (Invitrogen; PA5-44912) polyclonal antibodies were used at a 1:1,000 dilution. VTCN1 (Proteintech; 12080-1-AP) was used at a dilution of 1:500. The blots were incubated in their respective antibodies for 1 h at room temperature or overnight at 4°C. After 1 h, the blots were washed three times in PBST for 5 min. They were then placed in secondary antibody for 1 h (horseradish peroxidase [HRP]-linked goat anti-mouse or goat anti-rabbit [Pierce] antibodies were used at a 1:2,000 dilution). Blots were washed three times in PBST for 5 min before being incubated with ECL Western blot substrate. The blots were exposed to X-ray film (ThermoScientific) and processed in an X-Omat processor.

### Illumina sequencing.

Integrity of RNAs was assessed using an Agilent 2100 Bioanalyzer. cDNA was synthesized using reagents from the TruSeq RNA sample preparation kit (Illumina) according to the manufacturer's instructions. cDNA libraries were sequenced with a 73-base single-end read on an Illumina Genome Analyzer IIx at the Glasgow Polyomics facility at the University of Glasgow.

### Computational analysis.

Data sets were cleaned of reads with runs of >12Ns. Alignment to the human cDNA set (145,786 cDNAs—downloaded on 28 November 2011) was performed using Bowtie version 0.12.7. Further alignment to an updated human cDNA set (180,654 cDNAs downloaded 30 April 2012) was carried out using BWA 0.7.12-r1039. DESeq implemented in BioConductor (68) was used to select cellular genes whose expression was up- or downregulated by HPV in NIKS16 compared to NIKS cells implemented in the R environment. The raw read counts were normalized using reads per kilobase of transcript per million mapped reads (RPKM). DESeq uses a negative binomial error distribution to model transcript abundance and determine the differential expression. The significance of differential expression was estimated for each gene and then corrected for multiple comparisons (Padj). The top 1,000 differentially expressed genes based on log-fold change (Log_2_FoldChange) of >1.8 (3.5-fold change) are listed in Table S3 in the supplemental material.

### Functional analysis of differentially expressed genes.

GO (69) and KEGG (70) enrichment analyses were performed using Cytoscape (http://cytoscape.org/) with ClueGO (version 2.3.2) (71). The statistical test used for the enrichment was based on a two-sided hypergeometric option with a Bonferroni step-down correction, a *P* value of less than 0.05, and a kappa score of 0.4.

### Accession number(s).

Samples have been submitted to SRA@ncbi.nih.gov under the following Bioproject (GenBank) accession numbers: for the study, accession no. PRJNA379358 (SRP104232); for the sample NIKS16_D11_Mar17, accession no. SRS2131727; for the experiment with differentiated NIKS16 cells, accession no. SRX2745325; and for the run with NIKS_HPV16_D11_Mar17.fq.gz, accession no. SRR5457256. For the sample NIKS16_D5_Mar17, accession no. SRS2131728; for the experiment with undifferentiated NIKS16 cells (SRX2745326); and for the run with NIKS_HPV16_D5_Mar17.fq.gz, accession no. SRR5457258. For the sample NIKS_D11_Mar17, accession no. SRS2131729); for the experiment with differentiated NIKS cells, accession no. SRX2745327; and for the run with NIKS_D11_Mar17.fq.gz, accession no. SRR5457259. For the sample NIKS_D5_Mar17, accession no. SRS2131730; for the experiment with undifferentiated NIKS cells, accession no. SRX2745328; and for the run with NIKS_D5_Mar17.fq.gz, accession no. SRR5457260.

## Supplementary Material

Supplemental material
