# Supplementary material for: RNA-Seq Analysis of Differentiated Keratinocytes Reveals a Massive Response to Late Events during Human Papillomavirus 16 Infection, Including Loss of Epithelial Barrier Function
Source: J Virol. 2017 Nov 30;91(24):e01001-17. doi: 10.1128/JVI.01001-17 (PMC5709591; doi:10.1128/JVI.01001-17)
Supplement: Supplemental material [file supp_91_24_e01001-17__index.html]

Supplemental material 

# RNA-Seq Analysis of Differentiated Keratinocytes Reveals a Massive Response to Late Events during Human Papillomavirus 16 Infection, Including Loss of Epithelial Barrier Function

## Supplemental material

- Supplemental file 1 -

  Table S1 (Top 966 up- and downregulated genes.)

  XLSX, 84K
